# Influence of surfactant HLB values and commercial agricultural adjuvants on pesticide mimic penetration in plant leaves

**DOI:** 10.1002/ps.70591

**Published:** 2026-01-29

**Authors:** Begüm Demirkurt, Maarten Klein, Daniel Bonn

**Affiliations:** ^1^ Institute of Physics University of Amsterdam Amsterdam The Netherlands; ^2^ GreenA B.V. Amsterdam The Netherlands

## Abstract

**BACKGROUND:**

Effective pesticide action is crucial for optimizing efficacy and minimizing environmental impact, particularly with the increasing reliance on systemic pesticides. Adjuvants, including surfactants are commonly used to enhance penetration, but their performance depends on the physicochemical properties of both the pesticide and the surfactants used in the formulations. This study examines how surfactant hydrophilic–lipophilic balance (HLB) values and commercial adjuvants affect pesticide penetration through plant cuticles.

**RESULTS:**

We first assessed the penetration of two fluorescent pesticide mimics, Rhodamine B (hydrophilic) and Nile Red (lipophilic), into spring onion leaves using confocal laser scanning microscopy. On this highly waxy leaf, high HLB surfactants significantly enhanced the penetration of Rhodamine B, while low HLB surfactants promoted Nile Red penetration. Surfactants with intermediate HLB values had minimal effect on either compound. Among seven commercial adjuvants tested, only Squall and Prolong significantly improved the penetration of both pesticide mimics. To evaluate the role of leaf surface properties, we extended adjuvant experiments to sugarbeet and potato leaves, which have higher surface polarity and roughness. On these leaves, both fluorescent pesticide mimic penetrations (in the absence of any additive) were higher, and multiple adjuvants presented enhanced penetration, in contrast to their limited performance on green onion.

**CONCLUSION:**

Surfactant polarity plays a key role in overcoming cuticular resistance on waxy, low‐polarity leaves like green onion. On more permeable leaves, adjuvant performance is governed by surface characteristics. These findings underscore the importance of tailoring adjuvant selection to both pesticide polarity and crop‐specific leaf properties. © 2026 The Author(s). *Pest Management Science* published by John Wiley & Sons Ltd on behalf of Society of Chemical Industry.

## Graphical abstract

Penetration of a pesticide mimic into a spring onion leaf due to the presence of a surfactant additive.

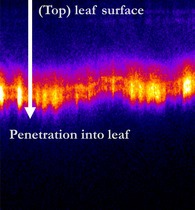

## 1 INTRODUCTION

Pesticides play a crucial role in modern agriculture, helping to control pests and diseases that can significantly reduce crop yields.^1^ Understanding the factors that influence pesticide penetration into plant tissues is essential for optimizing their application and minimizing environmental impact.^2^ Pesticides can be broadly categorized into systemic and contact types based on their mode of action and penetration ability.^3^

Systemic pesticides are absorbed by plants and transported throughout their tissues, including stems, leaves, roots, and flowers.^4^ In contrast, contact pesticides (non‐systemic) are absorbed to varying degrees by leaf tissues but show little to no translocation within plant vascular systems, remaining primarily localized at the application site.^5^ This characteristic allows systemic pesticides to provide long‐term protection against pests, even protecting new plant growth.^6^ They are particularly effective against diseases caused by various pathogens (fungi, bacteria, etc.) and pests (such as insects that may vector diseases) as these pesticides can spread internally through the plant, offering protection even when new growth occurs or when the pathogen is not directly exposed to the surface.^7^, ^8^ In contrast, contact (non‐systemic) pesticides act locally at the plant surface and exhibit little to no translocation.^8^ Their effectiveness depends on direct contact with target organisms. These pesticides are primarily suitable when immediate control or knockdown of pests is required, but they may not provide long‐term protection.^9^ The choice between systemic and contact pesticides depends on various factors, including the target pest, crop type, and environmental considerations.^4^

Among different diseases which need control to protect crop yield, potato late blight infection, a fungal disease caused by *Phytophthora infestans* is agronomically one of the most important.^10^ It is one of the most devastating diseases of potato, and one of the most destructive plant diseases across all major crops. Traditionally, multi‐site contact fungicides have been used to control the disease. Mancozeb, long favored for its affordability and broad‐spectrum activity, has been banned in the European Union due to concerns over its environmental impact and potential health risks. As a result, there is a shift towards single‐site systemic fungicides, which offer more targeted activity and improved performance under modern application constraints.^11^ Unlike contact fungicides, the systemic ones penetrate into the plant tissues, offering extended protection even in untreated areas, and are less prone to wash‐off by rain.^10^

Moreover, regulatory changes in pesticide application have introduced new challenges. Over the last decade, the use of anti‐drift nozzles has become mandatory in many agricultural settings.^12^ These nozzles, designed to reduce pesticide drift, produce larger droplets, which results in reduced spray coverage. For example, while traditional flat‐fan nozzles operating at standard pressures (2–3 bar) generate approximately >70 droplets per square centimeter, anti‐drift nozzles (such as 90% drift reduction types) operating at similar pressures produce ~20–40 droplets per square centimeter.^13^ This reduction significantly hampers the efficacy of contact fungicides, which rely on thorough surface coverage to be effective. In contrast, systemic fungicides are less dependent on droplet density, as their ability to penetrate plant tissues and spread internally means they can maintain efficacy even under reduced coverage conditions.

While the systemic fungicides deliver effective disease control, they come at a higher cost.^14^ The higher price of single‐site systemic fungicides contributes to increased crop protection expenses, underscoring the need for strategies that can enhance their penetration performance. In this context, adjuvants, especially surfactants, have emerged as vital tools. By improving the penetration of systemic fungicides into plant leaves, adjuvants can enhance the penetration and distribution of systemic products when properly matched to the active ingredient properties, helping to reduce the required dose and improve overall application efficiency. Some commercial agricultural adjuvants containing surfactants, like Squall, have been shown to improve the performance of crop protection products by helping active ingredients reach their targets more effectively and enhancing rainfastness.^15^

Penetration of pesticides into plant leaves is influenced by numerous factors, including the physicochemical properties of the pesticide, the characteristics of the plant cuticle, and environmental conditions.^2^ The plant cuticle, a waxy layer on the leaf surface, serves as a significant barrier to pesticide penetration.^16^, ^17^ Several techniques have been developed to assess pesticide penetration, including Surface‐Enhanced Raman Scattering (SERS) mapping, which allows for *in situ* and real‐time tracking of pesticides using gold nanoparticles as probes.^18^ Studies using SERS have shown that systemic pesticides penetrate more rapidly and deeply into live leaves compared to harvested leaves, with the systemic pesticide thiabendazole reaching depths of 225 *μm* in live leaves after 48 h of exposure.^18^

Adjuvants, particularly surfactants, play a critical role in enhancing pesticide efficacy by improving their penetration into plant tissues.^19^, ^20^ Surfactants (a class of adjuvants) are classified based on their hydrophilic–lipophilic balance (HLB) value, which influences their interaction with different types of pesticides and plant surfaces.^21^ Previous research has demonstrated that hydrophilic surfactants with high HLB values are most effective at enhancing the penetration of herbicides with high water solubility, whereas lipophilic surfactants with low HLB values are better suited for herbicides with low water solubility.^22^, ^23^ The mechanisms by which surfactants enhance pesticide penetration differ based on their HLB values. High HLB surfactants are absorbed into the cuticle and enhance the water‐holding capacity (hydration state) of the cuticle, which increases the permeance of hydrophilic pesticides.^22^, ^24^ In contrast, low HLB surfactants increase the fluidity of waxes in the cuticle, as measured by a small reduction in melting point, which enhances the permeance of lipophilic pesticides.^22^

Fungicide efficacy is increasingly intertwined with adjuvant science and resistance stewardship. Recent mechanistic work shows that non‐ionic alcohol ethoxylates with low ethylene oxide (EO) numbers markedly accelerate cuticular penetration of lipophilic triazoles without raising cuticular water loss;^25^ direct‐analysis mass spectrometry has confirmed that such surfactants transiently fluidize intracuticular waxes while leaving epicuticular structure intact.^26^ Field‐scale studies further demonstrate that essential‐oil‐based adjuvants can boost systemic insecticide and fungicide performance on high‐energy leaf surfaces, enabling 20–30% dose reductions.^27^ While single‐site systemic fungicides are increasingly favored due to regulatory constraints and improved performance under reduced droplet coverage conditions,^28^ they pose a higher risk of resistance development compared to multi‐site contact fungicides.^29^ This creates a challenging balance: systemic fungicides are more effective under current spray application constraints (such as mandatory anti‐drift nozzles), but their use must be carefully managed to prevent rapid resistance evolution. This situation underscores the importance of optimizing penetration efficiency through adjuvants, which could potentially allow for more sustainable use by improving efficacy at lower application rates. By improving penetration efficiency, adjuvants may potentially allow for optimized field application rates while maintaining efficacy,^30^, ^31^, ^32^, ^33^ though the relationship between dose and resistance selection pressure remains an active area of research.^32^, ^33^ While previous studies have examined the effects of surfactants on pesticide penetration,^34^, ^35^, ^36^ a systematic investigation using a wide range of HLB values with both hydrophilic and lipophilic pesticide mimics, coupled with an assessment of commercial adjuvants, has not been extensively documented in the literature. This study aims to fill this gap by investigating how surfactants with varying HLB values and commercial adjuvants affect the penetration of hydrophilic and lipophilic pesticide mimics into spring onion leaves, which is significantly difficult to wet due to highly waxy leaf surface.^37^, ^38^ To further assess the role of leaf surface characteristics, we also extended our study with commercial adjuvants to potato and sugarbeet leaves, which differ from spring onion in terms of waxy content, surface polarity, and roughness.^37^, ^38^, ^39^, ^40^, ^41^, ^42^, ^43^ Based on references,^37^, ^38^, ^39^, ^40^, ^41^, ^42^, ^43^ spring onions have approximately 15–20 μg cm^−2^ of epicuticular wax compared to potato leaves (8–12 μg cm^−2^) and sugar beet leaves (5–8 μg cm^−2^). The contact angles for water droplets are ~95° for spring onion, ~75° for potato, and ~ 65° for sugar beet, indicating decreasing hydrophobicity. Surface roughness measurements show spring onion has the smoothest surface (R_a_ ~ 0.5 μm) compared to potato (R_a_ ~ 1.2 μm) and sugar beet (R_a_ ~ 1.8 μm). We hypothesized that the higher wax content and lower surface polarity of spring onion would create a more selective barrier requiring polarity‐matched surfactants for effective penetration, while the less waxy, more polar, and rougher surfaces of potato and sugar beet would be more permeable to a broader range of adjuvants due to increased surface energy and mechanical irregularities that could facilitate penetration.

Our study builds on these insights by systematically mapping hydrophilic–lipophilic balance (HLB) effects across three crop species with contrasting cuticular barriers and tests the following hypotheses: (i) high HLB surfactants would enhance penetration of hydrophilic mimics while low HLB surfactants would improve lipophilic mimic penetration; (ii) commercial adjuvants (formulations containing surfactants) would show variable effectiveness based on their formulation properties; and (iii) leaf surface characteristics (wax content, polarity, roughness) would modulate adjuvant performance across different crop species.

To achieve this, confocal laser scanning microscopy (CLSM) was employed due to its widespread availability, non‐destructive nature, and excellent capability for imaging fluorescent pesticide mimics with high spatial and depth resolution. Several techniques have been developed to assess pesticide penetration into plant tissues. The Franz diffusion cell system provides quantitative permeation data using excised tissue but lacks spatial and real‐time resolution.^44^ LC–MS/MS employs a ‘spot and wash’ method to quantify residues but only offers bulk, endpoint measurements without spatial localization.^45^ SERS enables *in situ*, real‐time tracking with high sensitivity but requires specialized nanoparticles and equipment, limiting accessibility.^18^ In contrast, CLSM is widely available, non‐destructive, and well suited for imaging fluorescent pesticide mimics with high spatial and depth resolution.^34^ Additionally, CLSM allows the use of probes that mimic key pesticide properties, such as polarity, providing detailed mechanistic insights into penetration pathways and interactions within plant tissues.

## 2 MATERIALS AND METHODS

### 2.1 Pesticide mimics

Two fluorescent pesticide mimics were used in this study: Rhodamine B (hydrophilic) dissolved in water, and Nile Red (lipophilic) dissolved in a 50% (v/v) water/isopropanol mixture. These fluorescent dyes served as models for hydrophilic and lipophilic pesticides, respectively, allowing visualization of their penetration using confocal microscopy.^34^, ^35^, ^36^

### 2.2 Surfactants and commercial adjuvants

Five surfactants with systematically varying HLB values (Table 1), ranging from approximately 4 to 17, were selected to evaluate their effects on pesticide mimic penetration, which enable clear mechanistic insights into their mode of action.^21^ These included nonionic surfactants as they represent the vast majority of agricultural surfactants used commercially and avoid complications from ionic interactions with plant surfaces and environmental toxicity. All surfactants were prepared as 0.1% (v/v) aqueous solutions. In addition, the following commercial adjuvants commonly used in Dutch agriculture were tested: Prolong (P),^46^ Elasto (E),^47^ Wetcit Neo (W),^48^ Designer (D),^49^ CodaCide (C),^50^ WellPower (WP),^51^ and Squall (Sq).^52^ These commercial adjuvants were selected based on their widespread use in Dutch potato cultivation and, they represent a variety of structural types as detailed in Table 2, though their exact formulations are proprietary. It is worth noting that the HLB values provided in Table 2 are estimates based on the predominant surfactant types present in the formulations, as the exact compositions are not publicly disclosed. Commercial adjuvants were used according to the manufacturer's recommended field rates, with Squall applied at 0.5% (v/v) and all other adjuvants at 0.1% (v/v).

**Table 1:** Surfactants used in this study

Surfactant	Abbreviation	Chemical class	HLB
Tween 20	T20	Polyoxyethylene sorbitan ester	16.7
Tween 80	T80	Polyoxyethylene sorbitan ester	15
Brij O10	BO10	Polyoxyethylene oleyl ether	12.4
Span 20	S20	Sorbitan ester	8.6
Span 80	S80	Sorbitan ester	4.3

**Table 2:** Commercial adjuvants used in this study

Commercial adjuvant	Class	Chemical characteristics	Estimated HLB value	Rate used (v/v)	Manufacturer
Prolong	Nonionic surfactant	Alkylpyrrolidones, ligno‐sulfonates (micro‐emulsion)	8–12	0.1%	Profyto DSD, Oude Middenweg 231, 2491 AG Den Haag, The Netherlands
Elasto	Nonionic surfactant	Polyglycerol‐based	10–14	0.1%	SURfaPLUS B.V., Binnenhaven 1, 6709 PD Wageningen
Wetcit Neo	Anionic surfactant and essential oils	Sodium dodecylbenzenesulfonate based	12–14	0.1%	Rovensa Next, Avenue Louise 500, 1050 BRUSSELS, Belgium
Designer	Nonionic surfactant	Polyalkyleneoxide‐modified heptamethyltrisiloxane	1–6	0.1%	Nufarm B.V. Rivium, 2909 LC Capelle aan den Ijssel, The Netherlands
CodaCide	Essential oil and nonionic surfactant	Canola oil and polyethoxylated ester	6–10	0.1%	Microcide Ltd, Suffolk, IP31 2AR, United Kingdom
WellPower	Nonionic surfactant	Ethoxylated C11 alcohol	10–14	0.1%	Alpacor BVBA, Dijkstraat 16, 2800 Mechelen, Belgium
Squall	Nonionic surfactant	Polyethylene glycol	>15	0.5%	GreenA B.V., Science Park 301, 1098 XH Amsterdam, The Netherlands

### 2.3 Plant material and growing conditions

Three crop species were selected to represent a range of leaf surface characteristics commonly encountered in Dutch agriculture: spring onion (*Allium fistulosum* cv. Performer), potato (*Solanum tuberosum* cv. Agria), and sugar beet (*Beta vulgaris* cv. Leontine). The spring onion cultivar Performer is maintained by Bejo Seeds (Trambaan 1, 1749 CZ Warmenhuizen, The Netherlands). The potato cultivar Agria is maintained by Agrico B.V. (Duit 15, 8305 BB Emmeloord, The Netherlands). The sugar beet cultivar Leontine is maintained by KWS Saat SE & Co. KGaA (Grimsehlstr. 31, 37 574 Einbeck, Germany). All crops were grown under open‐field conditions on clay soil in commercial agricultural fields in the Netherlands, following standard agronomic practices typical for commercial production in the region. No additional irrigation was applied. Spring onions were harvested in April 2025 and harvesting was conducted in the morning between 09:00 and 12:00 under mild weather conditions, with ambient temperatures ranging from 5 to 10 °C, relative humidity between 55 and 75%, and predominantly cloudy conditions. Potatoes and sugar beets were harvested in June 2025 and harvesting was conducted in the morning between 09:00 and 12:00 under mild weather conditions, with ambient temperatures ranging from 10 to 20 °C, relative humidity between 55 and 75%, and predominantly cloudy conditions. Plants were harvested at well‐defined physiological growth stages to avoid senescence‐related effects. Spring onion plants were collected during the vegetative growth stage. Potato plants were sampled during the tuber bulking stage, with no visible signs of senescence. Sugar beet leaves were harvested during the root bulking stage. For experimental treatments, whole plants including roots were transported to the laboratory immediately after harvest. Healthy leaves were selected from the upper canopy to ensure consistent physiological status and surface characteristics. Leaves were used within 2 hours of harvest to preserve tissue viability and native leaf surface properties. For the experiments, three individual plants were harvested for potato and sugar beet and more than 10 plants for spring onion. Two to four leaves per plant were used. Leaves were randomly selected from these plants to minimize plant‐specific bias. The experimental design consisted of *n* = 2 biological replicates (different leaves from the same plant) and *n* = 3 technical replicates (distinct measurement areas per leaf), resulting in a total of six measurements per treatment. The plant selections provided a range of leaf surface characteristics: spring onion with high epicuticular wax content and smooth surfaces, potato with moderate wax content and intermediate surface roughness, and sugar beet with lower wax content and increased surface texture, allowing for comprehensive evaluation of adjuvant performance across different crop‐specific leaf barriers. Spring onion were chosen as the main sample for this study due to their waxy, difficult‐to‐wet leaves, which provide a challenging model for assessing pesticide penetration.^37^, ^38^ The use of plants with such characteristics would allow for a more rigorous evaluation of surfactant and adjuvant performance. Sugarbeet and potato leaves were also studied with the three commercial adjuvants (Squall,^52^ Designer^49^ and Wetcit Neo^48^) due to their different waxy contents in comparison to spring onion.^40^, ^41^, ^42^, ^43^

### 2.4 Penetration assessment using confocal microscopy

A drop of each surfactant or adjuvant solution containing the fluorescent pesticide mimic was applied to the surface of spring onion or other studied plant leaves. Solutions were applied using calibrated micropipettes (1 μL droplets) placed gently on the leaf surfaces. Application sites were chosen on flat areas avoiding major veins. After a 24‐h incubation period at room temperature (20–22 °C) and ambient lighting, excess solution was meticulously cleaned by using cotton buds wetted with 50% (v/v) water/acetone solution, and confocal laser scanning microscopy (CLSM) was employed to assess penetration depth (or diffusion length) from the leaf surface inward (from adaxial surface to abaxial).^53^ This approach allows for direct visualization of pesticide penetration, similar to methods used in previous studies investigating pesticide behavior in plant tissues.^34^, ^35^, ^36^ Our experimental design used *n* = 2 biological replicates (different leaves from the same plant) with *n* = 3 technical replicates (different measurement areas per leaf), giving a total of six measurements per treatment. The error bars represent standard deviation across all six measurements. We acknowledge this represents a limitation in biological replication and, future studies should include leaves from multiple plants to increase biological replication.

Fluorescence imaging was performed using a Leica DMi8 Inverted Confocal Scanning Laser Microscope equipped with a photomultiplier tube (PMT) detector. A 40× air objective (N PLAN L, NA 0.55, Leica) was used to acquire the images. The excitation wavelength was 552 nm for both dyes, and fluorescence emission was collected within the 575–700 nm spectral window. All leaf samples were scanned in the z‐direction over 300–400 μm (depending on the sample) with 1.3 μm step sizes for assessing the penetration/diffusion of the pesticide mimics. Cross‐sectional images were aligned side by side using the glass surface as the reference plane. Imaging parameters were optimized based on the sample with the lowest fluorescence response. To ensure comparability across different samples, the same imaging settings were used for all samples stained with the same dye, even if this led to saturation in some images.

All image processing and data analysis were performed using ImageJ and Python. Images were despeckled in ImageJ, and the FIRE LUT was applied to enhance contrast for better visualization of intensity variations. Mean diffusion length values (mean penetration depth) were calculated from thresholded cross‐section images. Global Otsu thresholding was applied, and the resulting binarized images were analyzed using a Python script. For each x‐coordinate in the cross‐section images, the script identified the last z‐coordinate with a value of 1, then averaged these values to determine the mean diffusion length for each sample.

## 3 RESULTS

### 3.1 Effect of surfactant HLB values

**Figure 1:**
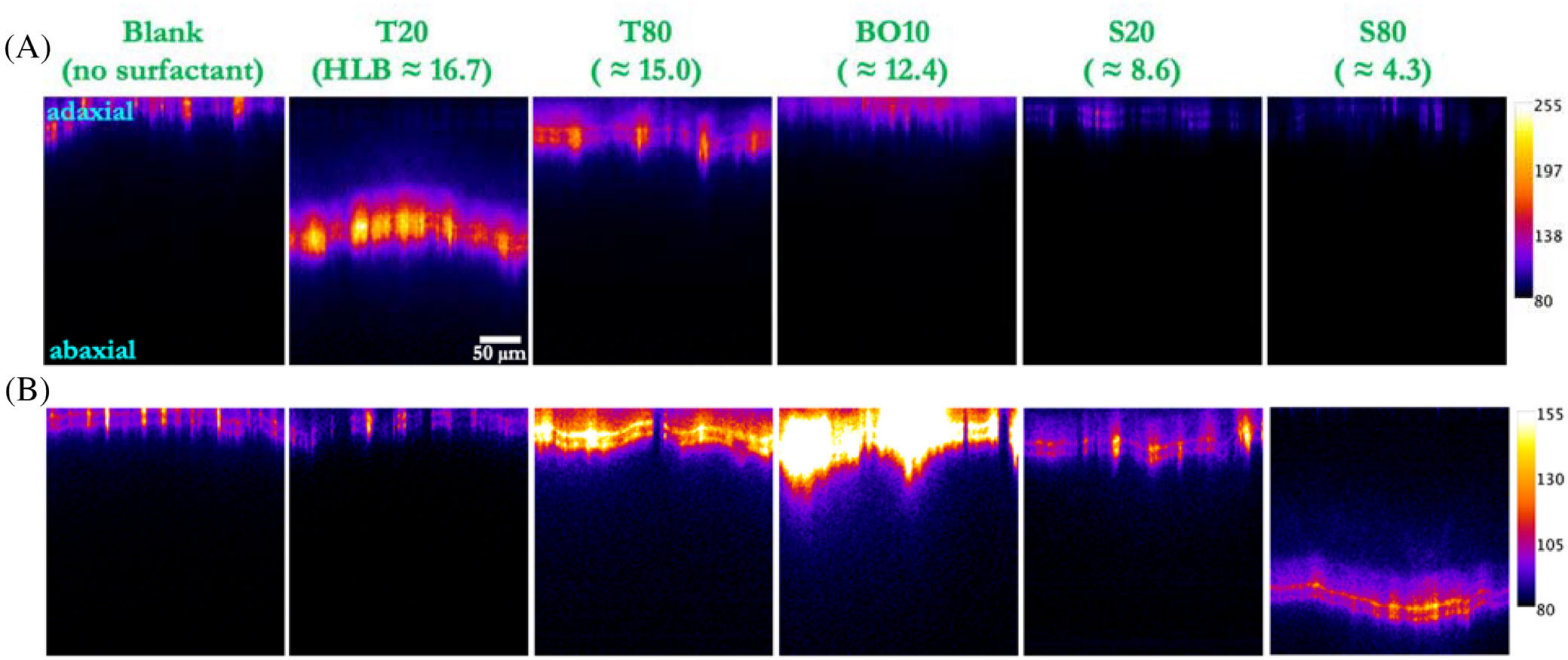
Penetration of (A) Rhodamine B (hydrophilic) and (B) Nile Red (lipophilic) through spring onion leaves in the presence (and absence) of different surfactants, 24 h after droplet application. All images show fluorescence intensity cross‐sections of the treated areas. The color scale represents fluorescence intensity values (8‐bit scale), where higher values correspond to brighter fluorescence.

The CLSM images revealed distinct patterns of penetration for the hydrophilic and lipophilic pesticide mimics in the presence of surfactants with varying HLB values, as presented in Fig. 1. High HLB surfactants (values approximately 15–22) significantly enhanced the penetration of the hydrophilic mimic Rhodamine B (Fig. 1(A)), while low HLB surfactants (values approximately 2–8) improved the penetration of the lipophilic mimic Nile Red (Fig. 1(B)).^22^, ^23^, ^35^

This observation aligns with previous research indicating that hydrophilic surfactants with high HLB values are most effective at enhancing the penetration of water‐soluble compounds, whereas lipophilic surfactants with low HLB values are better suited for compounds with low water solubility.^19^, ^22^ The enhancement of pesticide penetration by surfactants with extreme HLB values (either very high or very low) suggests that different mechanisms are involved in facilitating the penetration of hydrophilic and lipophilic compounds through the plant cuticle.^22^, ^24^

**Figure 2:**
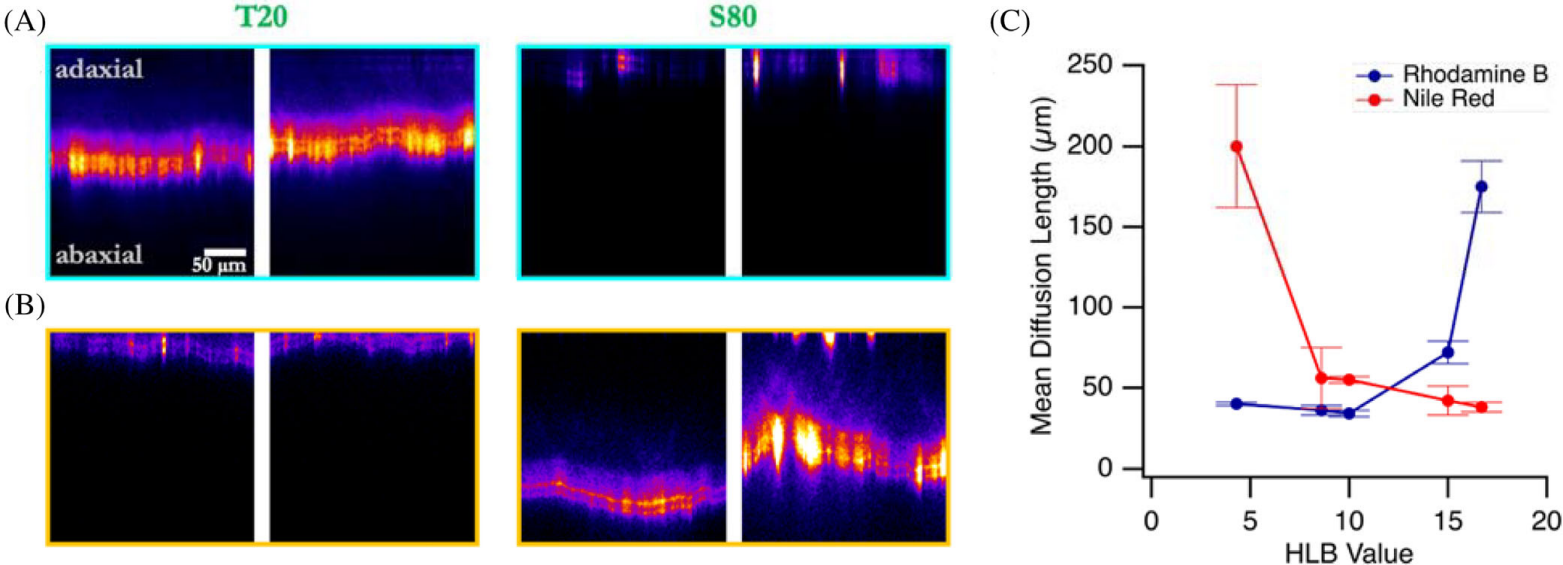
Fluorescence intensity cross‐section images showing the penetration of (A) Rhodamine B (hydrophilic) and (B) Nile Red (lipophilic) through spring onion leaves in the presence of Tween 20 and Span 80 surfactants, representing the two extremes of the HLB values studied, 24 h after droplet application. Images are taken across different areas of the treated leaves. (C) Mean diffusion lengths plotted against the HLB values of the surfactants added to the fluorescent mimic solutions. Error bars represent standard deviation across two leaves (biological replicates) and three areas per leaf (technical replicates).

Figure 2 shows that the mean penetration depth (or mean diffusion length) increased with increasing HLB value of the surfactant for Rhodamine B, with the highest penetration observed with surfactants having HLB values above 15. In contrast, Nile Red penetration was most enhanced by surfactants with HLB values below 8. Surfactants with intermediate HLB values (~9–12) showed limited enhancement of penetration for both mimics.

### 3.2 Effect of commercial adjuvants

**Figure 3:**
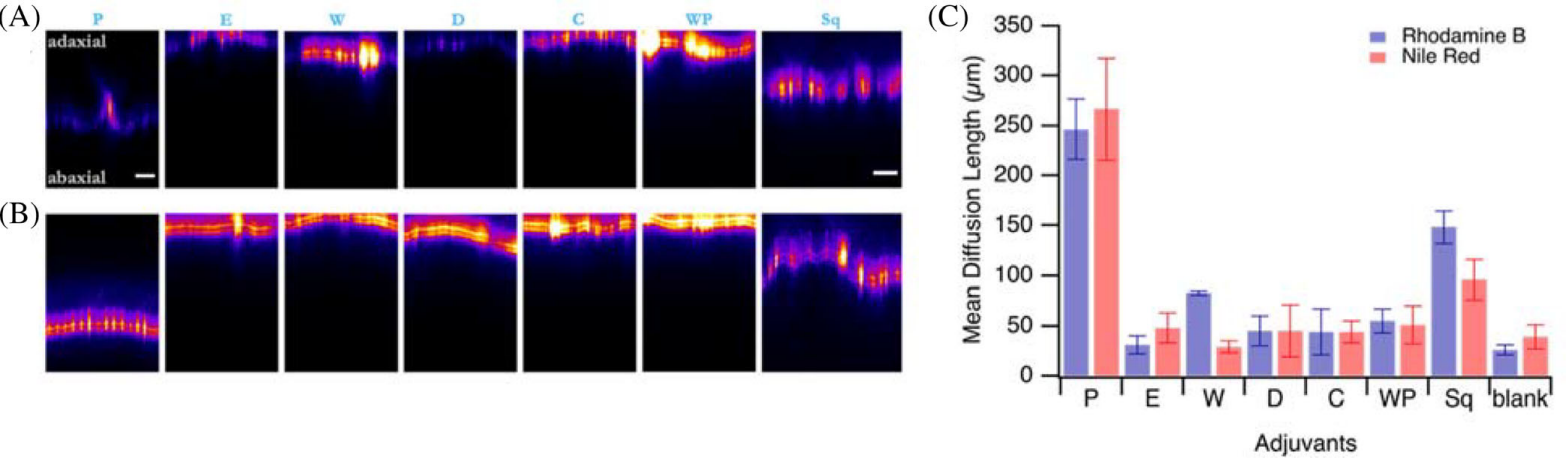
Fluorescence intensity cross‐section images showing the penetration of (A) Rhodamine B (hydrophilic) and (B) Nile Red (lipophilic) through spring onion leaves in the presence of commercial adjuvants, 24 h after droplet application. All scale bars represent 50 μm. (C) Mean diffusion lengths plotted against the different adjuvants added to the fluorescent mimic solutions. Error bars represent standard deviation across two leaves (biological replicates) and three areas per leaf (technical replicates).

Among the commercial adjuvants tested, only Squall^52^ and Prolong^46^ demonstrated significant enhancement of pesticide mimic penetration (Fig. 3). Other adjuvants did not significantly affect the penetration of either Rhodamine B or Nile Red into spring onion leaves.

The effectiveness of Squall aligns with its documented ability to improve the performance of crop protection products by helping active ingredients reach their targets more effectively.^52^ Squall has been shown to enhance rainfastness, with scientific field tests confirming that 50% more active ingredients remain on the crop after a standard rain shower when Squall is added to the tank mix.^52^

The limited effectiveness of most commercial adjuvants in enhancing pesticide penetration suggests that while they may improve spray coverage and retention, they may not necessarily facilitate the movement of pesticides through the highly waxy plant cuticle types. This finding highlights the importance of selecting adjuvants based on specific application requirements, rather than assuming that all adjuvants will enhance penetration.^24^

### 3.3 Other plant species: potato and sugarbeet

To investigate how adjuvant performance is influenced by the properties of different leaf surfaces, we extended our penetration experiments to sugarbeet and potato leaves, which differ from green onion in terms of cuticle morphology, polarity, and surface roughness.^37^, ^38^, ^39^, ^40^, ^41^, ^42^, ^43^ Using the same pesticide mimics and three selected commercial adjuvants (Squall,^52^ Designer,^49^ and Wetcit Neo^48^), we observed marked differences in penetration behavior across the three species.

**Figure 4:**
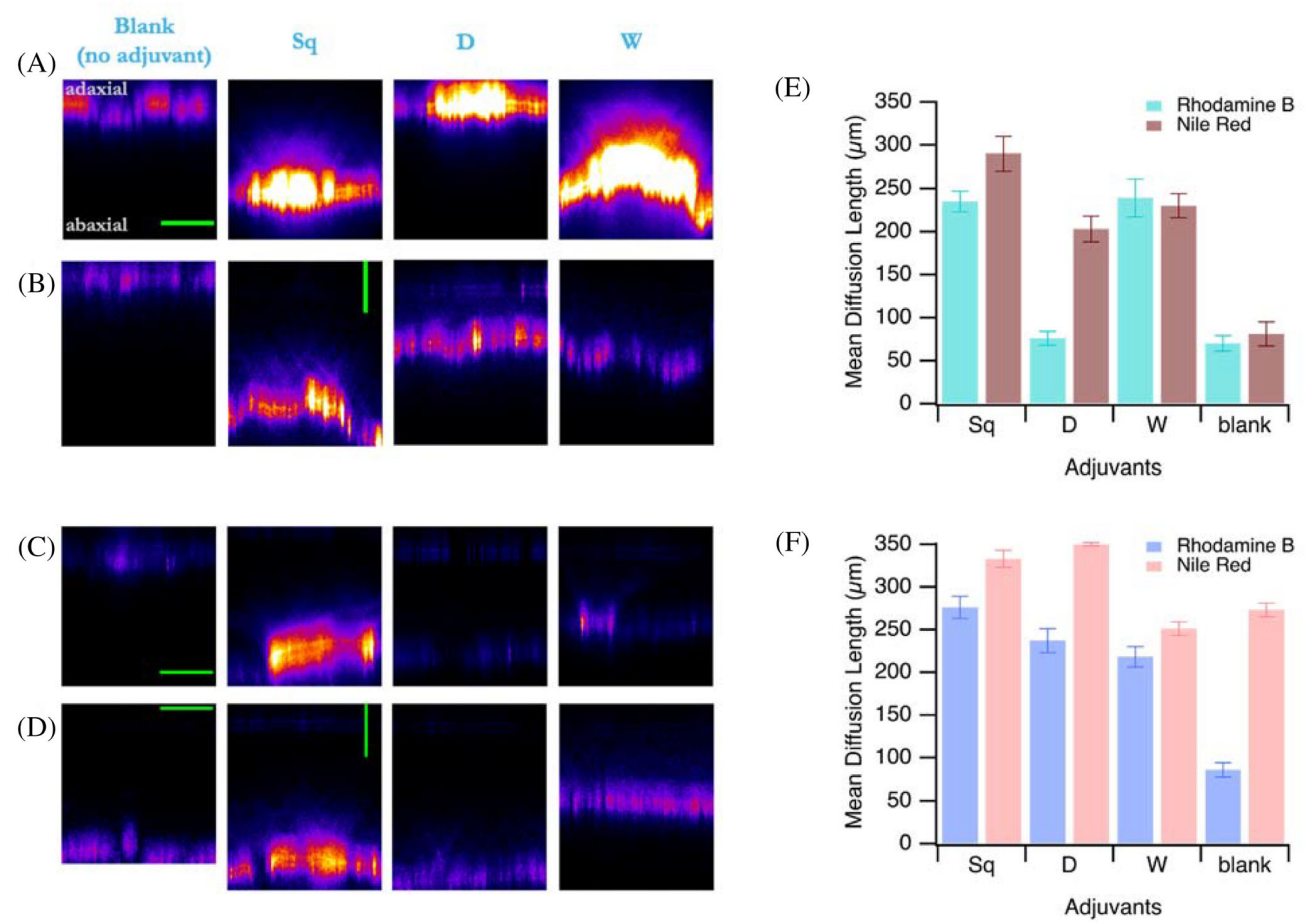
Penetration of Rhodamine B (hydrophilic) and Nile Red (lipophilic) through sugarbeet (A and B, respectively) and potato (C and D, respectively) leaves in the presence (and absence) of selected commercial adjuvants, 24 h after droplet application. All scale bars represent 100 μm. All images show fluorescence intensity cross‐sections of the treated areas. Mean diffusion lengths plotted against the different adjuvants added to the fluorescent mimic solutions for sugarbeet (E) and potato leaves (F). Error bars represent standard deviation across two leaves (biological replicates) and three areas per leaf (technical replicates).

In contrast to green onion, where only Squall^52^ (and Prolong^46^) significantly enhanced dye penetration, all three tested adjuvants led to notable increases in penetration depth for both Rhodamine B and Nile Red on sugarbeet and potato leaves (Fig. 4). Moreover, baseline penetration in the absence of adjuvants (blank) was measured to be higher for these two plant species, particularly in potato leaves, indicating inherently more permeable surfaces. Notably, Designer^49^ and Wetcit Neo,^48^ which were largely ineffective on green onion, showed substantial effectiveness on the penetration of both pesticide mimics in sugarbeet and potato leaves. Squall^52^ consistently produced strong enhancement across all studied species and dyes.

## 4 DISCUSSION

The results of this study provide important insights into the factors that influence pesticide penetration into plant leaves and the role of surfactants and commercial agricultural adjuvants in modulating this process across different plant species. On green onion leaves, which are highly waxy and difficult to wet,^37^, ^38^ we observed a strong dependence on surfactant polarity: high HLB surfactants enhanced the penetration of the hydrophilic mimic Rhodamine B, while low HLB surfactants improved penetration of the lipophilic mimic Nile Red (Figs 1 and 2(C)). This behavior aligns with established mechanisms in the literature, where high HLB surfactants increase the hydration capacity of the cuticle, facilitating hydrophilic compound transport, and low HLB surfactants fluidize cuticular waxes, thereby enhancing lipophilic compound permeability.^22^, ^23^, ^24^ It is worth noting that limited biological replication represents a constraint of the present study; nevertheless, consistent trends were observed across technical replicates for spring onion leaves selected from multiple individual plants. While effective penetration enhancement was observed with extreme HLB surfactants, it is important to note that such formulations can alter cuticular transpiration when applied at excessively high concentrations. However, these effects have been shown to occur only at surfactant loadings far beyond typical agricultural use rates, and are unlikely to pose practical risks under field‐relevant conditions.^25^ Among the commercial adjuvants tested, the effectiveness of Squall^52^ and Prolong^46^ in enhancing penetration for both pesticide mimics (Fig. 3) suggests that these adjuvants either have formulations with extreme HLB values or may incorporate other mechanisms that facilitate pesticide movement through the plant cuticle.

The extended experiments on sugarbeet and potato leaves demonstrate the importance of leaf surface properties in modulating cuticular resistance and adjuvant efficacy. On the highly waxy and smooth surface of green onion,^37^, ^38^ the combination of low polarity and minimal roughness^39^ likely presents a compact and hydrophobic barrier, requiring strong polarity‐matched surfactants to enable the penetration of pesticide mimics. In contrast, potato and sugarbeet leaves, with higher polarity and surface roughness,^40^, ^41^, ^42^, ^43^ appear more responsive to general spreading and hydration mechanisms, which can facilitate dye penetration even in the absence of tailored adjuvants, as we observed (Fig. 4). Squall^52^ consistently produced strong enhancement across all studied species and dyes. This robustness suggests that Squall^52^ may operate *via* multiple mechanisms or contain a balanced surfactant composition that is broadly effective across surface chemistries. Overall, these results highlight that optimal adjuvant selection requires careful consideration of not only the active ingredient's properties but also the specific surface characteristics of the target crop, particularly when systemic penetration is essential for efficacy.

This study demonstrates the practical utility of confocal microscopy as an accessible method for assessing pesticide penetration.^34^, ^35^, ^36^ While we did not perform direct statistical comparisons with alternative methods such as SERS mapping^17^, ^18^ or Franz diffusion cells,^44^ CLSM provided sufficient spatial and temporal resolution for our comparative analysis of surfactant and adjuvant effects.

In the broader context of pesticide use in agriculture, the findings of this study have important implications for the ongoing shift from contact to systemic pesticides.^3^, ^6^ Systemic pesticides have been favored for their ability to provide long‐term protection by being absorbed and distributed throughout the plant.^4^, ^7^ However, concerns about their environmental impact, particularly on non‐target organisms, have led to increased scrutiny.^28^ Enhancing the penetration of pesticides through the strategic use of adjuvants, including surfactants, could potentially allow for lower application rates, thereby reducing environmental impact while maintaining efficacy.^25^, ^32^, ^33^

## 5 CONCLUSION

This study demonstrates that the hydrophilic–lipophilic balance (HLB) of surfactants plays a critical role in enhancing pesticide penetration into plant leaves, particularly on surfaces with high cuticular resistance. On waxy and smooth leaves like green onion, high HLB surfactants significantly improved penetration of hydrophilic compounds, while low HLB surfactants were more effective for lipophilic compounds. However, this polarity‐matching effect was less pronounced on sugarbeet and potato leaves, where increased surface polarity and roughness allowed for broader adjuvant responsiveness and higher baseline permeability.

Among the tested commercial adjuvants, Squall consistently enhanced penetration across all leaf types and dye chemistries, suggesting a formulation that is effective under varied surface conditions. Designer and Wetcit Neo, though ineffective on green onion, showed substantial enhancement on potato and sugarbeet, further emphasizing the importance of leaf surface characteristics, such as wax load, in determining adjuvant performance.

These findings suggest that optimal adjuvant selection likely requires consideration of both active compound properties and crop‐specific leaf surface characteristics, as inferred from literature values for wax content, surface polarity, and roughness. Direct measurement of leaf surface properties in future studies would strengthen this conclusion.

The findings of this study contribute to our understanding of how adjuvants (including surfactants) influence pesticide behavior in plant systems and provide valuable insights for optimizing pesticide formulations and application strategies. Future research should focus on further elucidating the mechanisms by which surfactants enhance pesticide penetration and on developing more effective adjuvant formulations that balance efficacy with environmental safety.

## Data Availability

The data that support the findings of this study are available from the corresponding author upon reasonable request.
